# Finite Element Modeling of the Thermal Conductivity of Polymer Composites Reinforced with Graphite Sheets

**DOI:** 10.3390/polym18121445

**Published:** 2026-06-10

**Authors:** Abdulrahman A. Alghamdi

**Affiliations:** Mechanical Engineering Department, College of Engineering and Architecture, Umm Al-Qura University, Makkah, Saudi Arabia; aasghamdi@uqu.edu.sa; Tel.: +966-555531140

**Keywords:** thermal conductivity, finite element modeling, epoxy/graphite sheet, polymer matrix composite

## Abstract

Efficient heat dissipation represents a critical challenge for maintaining device performance and reliability in modern electronic devices. Polymer composites reinforced with graphite sheets have attracted attention as thermal interface materials because of their lightweight nature and excellent thermal transport properties. Herein, the effects of graphite sheet volume fraction, sheet thickness, folding angle, and sheet orientation on the through-thickness thermal conductivity of epoxy/graphite sheet composites were investigated using finite element (FE) modeling. A reduced folding angle and increased graphite sheet thickness enhanced the through-thickness thermal conductivity. However, for the same graphite volume fraction, reducing the folding angle enhanced thermal conductivity more effectively than increasing the graphite sheet thickness, indicating the dominant role of sheet orientation in the heat transport behavior. The influence of the folding angle became significant at higher graphite volume fractions due to the formation of more continuous conductive pathways. At a graphite sheet volume fraction of 0.5, the thermal conductivity decreased from 22.27 to 11.52 W m^−1^ K^−1^ upon increasing the folding from 15° to 90°. Finally, a semi-empirical model exhibiting good agreement with the FE results was developed, demonstrating that optimization of graphite sheet geometry is essential for improving the thermal performance of polymer-based thermal interface materials.

## 1. Introduction

The rapid development of small, powerful, and high-frequency electronic devices has increased the demand for effective heat-dispersion materials. As components shrink and become more compact, local overheating can reduce device efficiency, accelerate material degradation, and shorten service life [1,2]. Thermal management materials are therefore essential for transferring heat away from electronic components such as power modules, light-emitting diodes, and batteries. Among these materials, thermal interface materials (TIMs) play a key role by reducing interfacial thermal resistance and facilitating heat transfer between heat sources and sinks [3,4]. However, standard polymer-based TIMs tend to exhibit low thermal conductivity (i.e., 0.1–0.5 W m^−1^ K^−1^), thereby limiting their use in high-performance cooling systems [5]. This challenge has driven research on thermally conductive polymer composites.

In the context of thermal management applications, polymer composites offer several advantages, including low density, corrosion resistance, ease of processing, good mechanical flexibility, and design versatility [6,7]. Epoxy resins are the most widely used polymer matrices due to their excellent adhesion, chemical resistance, dimensional stability, and compatibility with electronic packaging processes [8]. However, because of its low thermal conductivity, epoxy resin requires reinforcement with thermally conductive fillers to form effective heat-transfer pathways [5]. Various fillers have been investigated to date, including ceramic particles, such as alumina, aluminum nitride [9], boron nitride, and silicon nitride [10]. Carbon-based fillers such as graphite [11], graphene, carbon nanotubes, and carbon fibers have also been studied [12].

Although ceramic fillers are often preferred to impart electrical insulation, carbon-based fillers are attractive because of their high thermal conductivity and lightweight structures [13]. For instance, graphene and graphite possess high in-plane thermal conductivities owing to strong covalent bonds and efficient within-layer phonon transport [14]. Consequently, graphite and graphene nanoplatelets and sheets are widely used to enhance the thermal conductivity of polymers. Yu et al. [15] demonstrated the potential of graphite nanoplatelets as thermally conductive fillers for epoxy-based thermal interface materials. Shahil and Balandin [16] showed that graphene and multilayer graphene can significantly improve heat transport in polymer composites because of their high intrinsic thermal conductivity. Song et al. [17] reviewed the role of two-dimensional materials in thermal management applications, highlighting their ability to form efficient conductive networks. Fu et al. [18] further discussed graphene-related materials for thermal management and emphasized the importance of filler orientation, dispersion, and interface quality in achieving high composite thermal conductivity.

Studies have shown that graphite- and graphene-based fillers improve the conductivity of epoxy composites by forming networks within the polymer matrix [15,16]. Nevertheless, achieving high conductivity requires not only a high filler content, but also an appropriate filler orientation, good dispersion, strong interfacial contact, and continuous heat-conducting pathways [19]. However, poor dispersion, clumping, voids, and interfacial resistance can significantly reduce composite conductivity, even in the presence of suitable fillers [20,21].

The development of efficient through-thickness heat-transfer pathways represents a major challenge in the field of thermally conductive polymer composites. Many carbon-based fillers exhibit highly anisotropic thermal conductivity, with heat transfer being significantly greater along the in-plane direction than through the thickness direction [13,14]. Consequently, composites incorporating randomly dispersed or horizontally aligned graphite or graphene fillers may demonstrate significantly enhanced in-plane thermal conductivity, while showing only limited improvement in the through-thickness direction. In TIM applications, the through-thickness thermal conductivity is often more important, as heat must be transferred across the material thickness from the heat source to the heat sink [22]. Therefore, structural design strategies that orient graphite sheets or graphene networks along the heat-flow direction are essential for improving through-thickness thermal transport properties.

Several approaches have been proposed for the construction of continuous thermally conductive networks in polymer composites. These include filler alignment under external fields, freeze casting, compression-induced orientation, three-dimensional porous networks, hybrid fillers, and preformed carbon architectures. Xie et al. [22] compared the thermal conductivity enhancement of graphene nanosheets and carbon nanotubes, highlighting the importance of filler geometry and connectivity. Mortazavi et al. [23] demonstrated through multiscale modeling that heat transfer in graphene/epoxy composites is strongly affected by filler distribution and interfacial thermal resistance. Razzaghi et al. [24] further highlighted that numerical modeling can help clarify the relationship between filler arrangement, thermal pathways, and effective composite thermal conductivity. These studies indicate that the formation of continuous and well-oriented conductive networks is more important than simply increasing the filler content.

The graphite sheet architecture is a promising strategy. Specifically, graphite sheets can provide continuous, highly conductive pathways while maintaining a relatively low filler content compared with randomly dispersed particles. In this context, Li et al. [25] reported polymer composites reinforced with polypropylene-folded graphite paper, demonstrating significantly enhanced thermal conductivity. Such architectures are attractive because the heat-transfer performance depends not only on filler content but also on geometric parameters. These parameters include the sheet thickness, folding angle, orientation, and structural periodicity.

The objective of this study is therefore to investigate the effects of the above parameters on the through-thickness thermal conductivity of epoxy resin/graphite sheet (EP/GS) composites using the finite element (FE) method. Subsequently, the FE results are validated against previously reported experimental results. Furthermore, a semi-empirical model combining the anisotropic conductivity of the graphite sheets with a modified rule of mixtures is developed based on the FE results.

## 2. Materials and Methods

### 2.1. Materials

**Figure 1:**
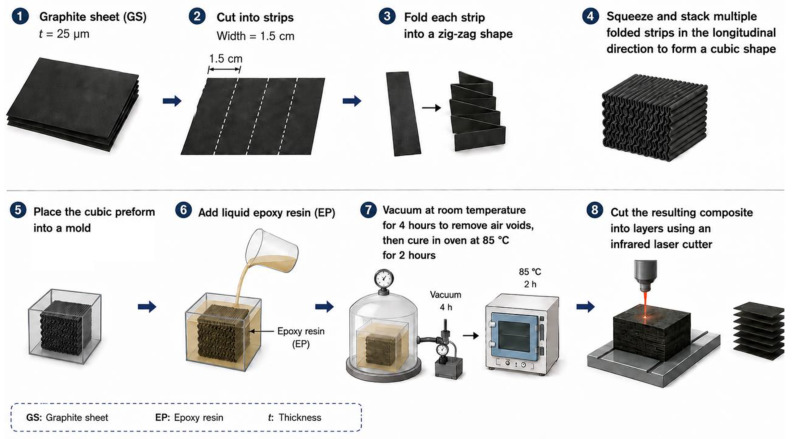
Preparation of the EP/GS composite.

**Figure 2:**
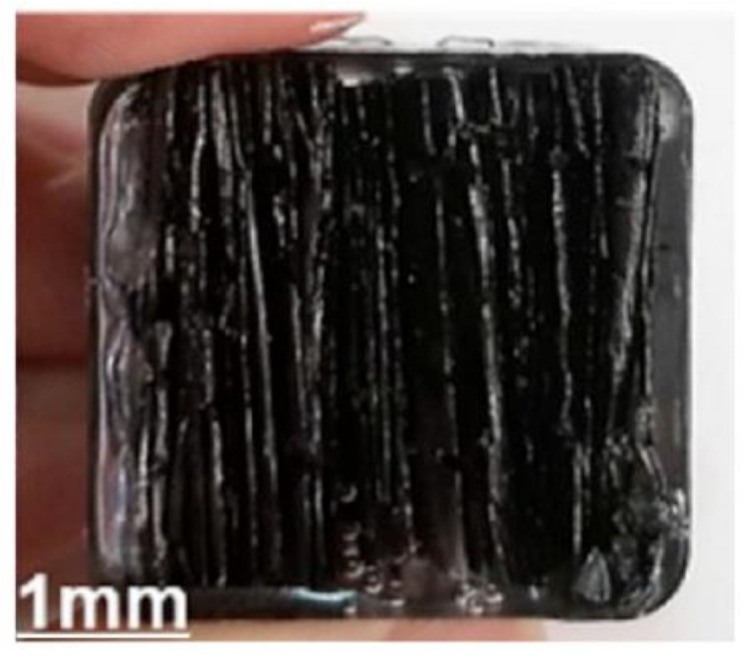
The EP/GS composite [25].

The composite investigated in this study was a polymer matrix composite (PMC) consisting of epoxy resin and graphite sheets, fabricated by Li et al. [25]. The graphite sheets (25 µm thickness) were cut into 1.5 cm strips and then folded into zigzag shapes. Multiple folded strips were squeezed and stacked along the longitudinal direction to produce cubes, which were placed in a mold, and liquid epoxy resin was added. Subsequently, the mold was subjected to vacuum at room temperature for 4 h to remove air voids, then cured in an oven at 85 °C for 2 h. The resulting composites were cut into layers using an infrared laser cutter [25]. Figure 1 outlines the process employed to prepare the EP/GS composites, while Figure 2 shows a photographic image of the composite material.

### 2.2. Finite Element Modeling

**Figure 3:**
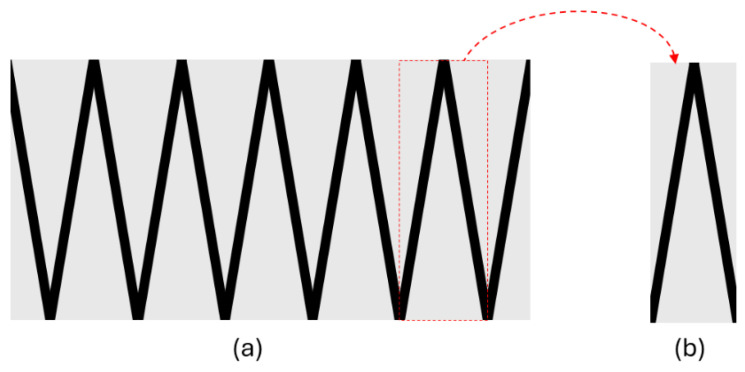
(**a**) The periodically repeated zigzag structure of the EP/GS composite, and (**b**) the EP/GS composite unit cell.

FE modeling was employed to predict the through-thickness thermal conductivity of the EP/GS composites and investigate the influence of various geometric and material parameters on the resulting heat-transfer behavior. The modeling of composite materials can be simplified by considering either a representative volume element (RVE) or a unit cell. An RVE represents heterogeneous materials whose microstructures are macroscopically or statistically homogeneous over sufficiently large scales, whereas a unit cell models heterogeneous materials with periodic microstructures [26,27]. In this study, the folded graphite sheets within the EP/GS composite exhibited a periodically repeated zigzag configuration (Figure 3a). FE analysis was therefore simplified by modeling a representative unit cell of the composite structure (Figure 3b). The complete composite architecture was then generated by periodically repeating the unit cell through its translation along three spatial directions, namely the (X)-, (Y)-, and (Z)-axes.

#### 2.2.1. Geometry of the EP/GS Composite Unit Cell

**Figure 4:**
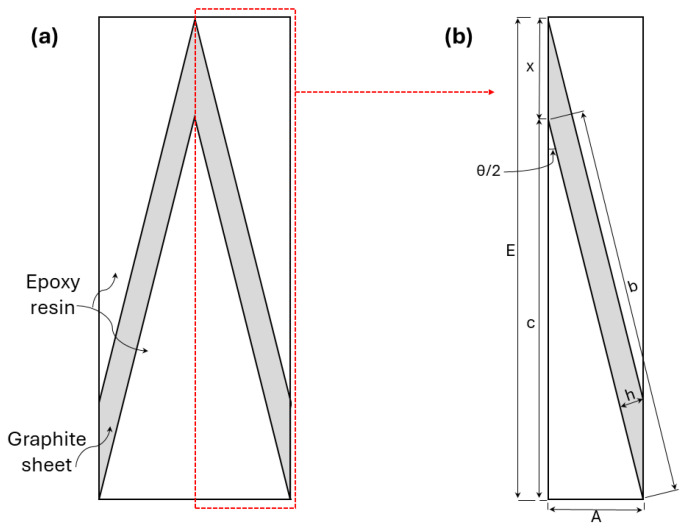
(**a**) Front view of the composite unit cells, and (**b**) the unit cell parameters.

Figure 4a shows a front view of the composite unit cells, while Figure 4b shows the unit cell parameters for half a cell. The following equations were derived to obtain the dimensions of the finite element unit cell from the known values of the graphite-sheet volume fraction ($V_{GS}$), folding angle (*θ*), and graphite-sheet thickness (*h*). Specifically, the volume fraction was calculated by dividing the area of the graphite sheet by the total area of the unit cell as follows:

(1) $$V_{GS}=\frac{h\times b}{A\times E}$$

The area of the graphite sheet equals the area of the gray parallelogram shown in Figure 4b, which can be calculated by multiplying *h* by the base of the parallelogram (*b*). Additionally, *b* can be calculated from *θ* and the half-width of the unit cell (*A*), as follows:

(2) $$b=\frac{A}{sin\;(\frac{\theta }{2})}$$

The height of the FE unit cell (*E*) is the sum of (*c*) and (*x*):

(3) E=c+x,

where *c* can be calculated from *θ* and *A* as follows:

(4) $$c=\frac{A}{tan\;(\frac{\theta }{2})}$$

Furthermore, *x* can be calculated from *θ* and *h* according to the following expression:

(5) $$x=\frac{h}{sin\;(\frac{\theta }{2})}$$

By applying Equations (2)–(5) to Equation (1), *A* can be obtained, as follows:

(6) $$A=\left(\frac{1}{V_{GS}}-1\right)\times \frac{h}{\cos\left(\frac{\theta }{2}\right)}.$$

In this study, the through-thickness thermal conductivity of the EP/GS composite was evaluated using a parametric approach based on the geometric unit cell shown in Figure 4. The main geometric parameters were the height *E*, half-width *A*, graphite sheet thickness *h*, and folding angle *θ*, while the graphite sheet volume fraction $V_{GS}$ was treated as a key variable. During the parametric study, two geometric parameters were simultaneously fixed, whereas the remaining geometric parameters and $V_{GS}$ were varied systematically. This procedure enabled the isolation of the effects of each design parameter on the effective through-thickness thermal conductivity. By comparing the results from different parameter combinations, the influence of the graphite sheet folding angle, sheet thickness, and structural dimensions on the heat-transfer performance was identified.

**Figure 5:**
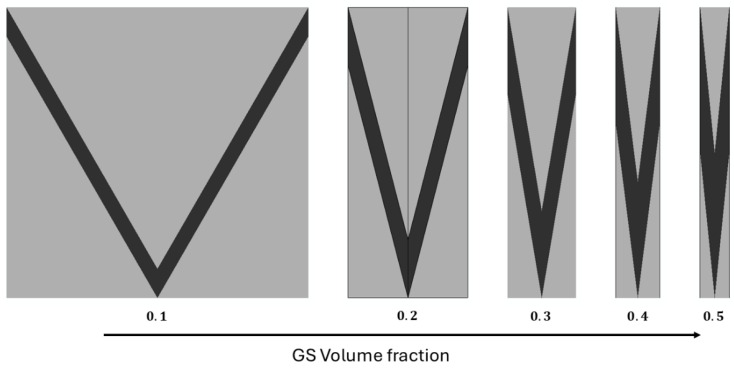
FE models based on a fixed height and GS thickness.

In the first case, five FE models, *E* and *h* were fixed at 0.5 mm and 25 µm, respectively, while the width (2A), θ, and $V_{GS}$ were varied. Figure 5 shows front views of these models.

**Figure 6:**
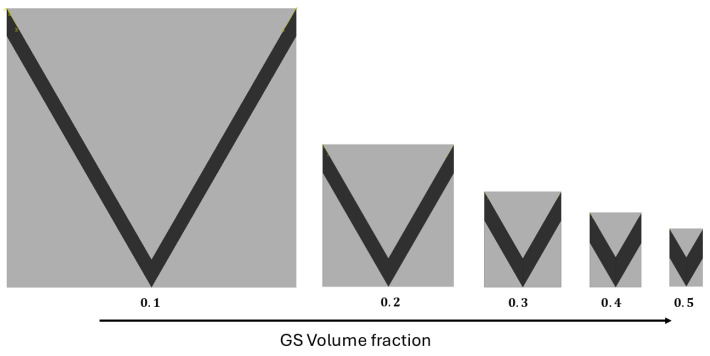
FE models with a fixed folding angle and GS thickness.

In the second case, six sets of models containing 30 FE models were constructed using fixed values of θ and *h*, together with variations in 2A, *E*, and $V_{GS}$. Specifically, *h* was fixed at 25 µm, while θ was increased from 15° to 90° in increments of 15° for each set. For example, in the first set of the second case, θ and *h* were fixed at 15° and 25 µm, respectively, while in the second set of the second case, θ and *h* were fixed at 30° and 25 µm, respectively. Figure 6 shows a front view of one of these sets.

**Figure 7:**
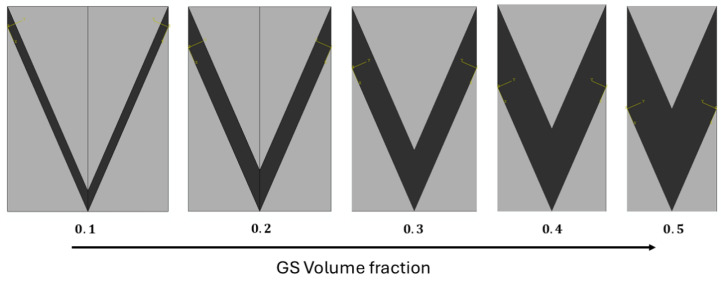
FE models with fixed height and folding angle.

In the third case, five FE models were constructed, wherein *E* and θ were fixed, whereas 2A, *h*, and $V_{GS}$ were varied. Figure 7 shows the front views of these models.

#### 2.2.2. Boundary Conditions and Material Properties

Based on Fourier’s law for one-dimensional steady-state heat conduction, the heat transfer rate between two isothermal surfaces is proportional to both the cross-sectional area normal to the heat flow direction and the applied temperature gradient, and can be expressed as follows:

(7) $$Q=-kA\frac{dT}{dx}$$

where *Q* denotes the heat transfer rate, *k* is the thermal conductivity, *A* represents the cross-sectional area perpendicular to the heat flow direction, and $\frac{dT}{dx}$ is the temperature gradient.

**Figure 8:**
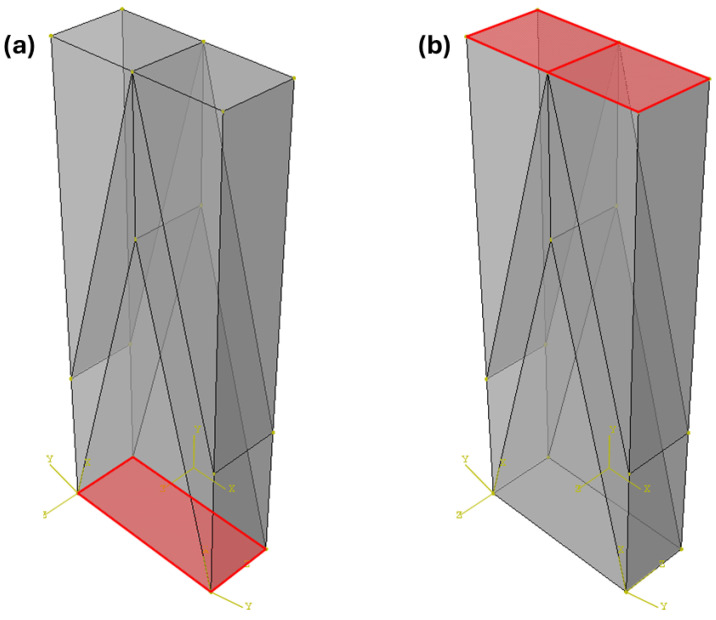
Boundary conditions in a transparent composite unit cell for (**a**) the low-temperature surface, and (**b**) the high-temperature surface.

The effective thermal conductivity of the FE model was evaluated through steady-state thermal analysis. In the FE simulation, the thermal boundary conditions were defined by assigning two different temperatures, $T_{High}$ and $T_{Low}$, to the two opposite surfaces of the model, whereas the remaining surfaces were assumed to be thermally insulated. The imposed temperature difference generated a constant heat flux from the high-temperature surface to the low-temperature surface. The resulting heat flux *Q*, obtained from the post-processing module of the FE software (ABAQUS, version 6.14 [28]), was applied to Fourier’s law to calculate the effective thermal conductivity of the model according to Equation (8):

(8) $$k=-\frac{Q\times L}{A\times (T_{High}-T_{Low})}$$

where *k* is the effective thermal conductivity of the model, *Q* is the heat flux through the low-temperature surface, *L* is the model thickness in the heat-flow direction, *A* is the cross-sectional area, and $T_{High}$ and $T_{Low}$ are the temperatures applied to the hot and cold surfaces, respectively. Figure 8 illustrates the boundary conditions of a transparent composite unit cell. Specifically, Figure 8a shows the surface with the low-temperature surface, whereas Figure 8b shows the low-temperature and high-temperature surfaces, respectively.

**Figure 9:**
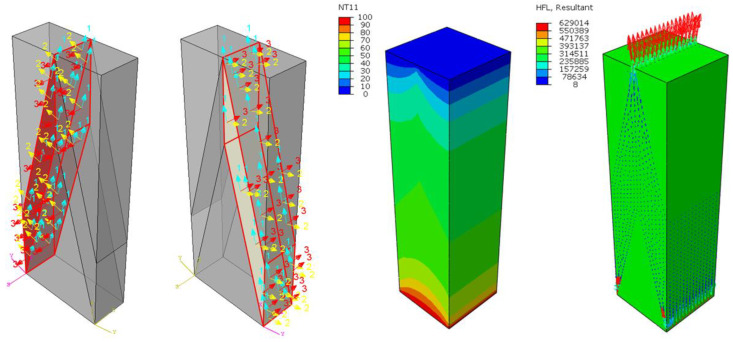
In-plane and through-thickness directions of the graphite sheet and the temperature and heat flux contours.

Table 1 lists the assigned material properties of the epoxy resin and the graphite sheet. Due to the directionality of the graphite sheet, a rectangular data coordinate system was created on each side of the sheet. Figure 9 shows the in-plane and through-thickness directions of the graphite sheet, where 2 refers to the through-thickness direction and 1 and 3 refer to the in-plane direction.

## 3. Results and Discussion

### 3.1. Validation of the FE Model

The experimental results reported by Li et al. [25] were used to validate the FE modeling approach developed herein. In their study, the through-thickness thermal conductivity of the EP/GS composite was measured as a function of the graphite sheet volume fraction. However, the volume fraction is influenced by several geometric parameters, including the sheet thickness, folding angle, and mold size. Therefore, the FE model developed for validation was designed to reproduce the experimental configuration as closely as possible. Since the graphite sheet thickness and mold dimensions were maintained constant in the experimental setup, the folding angle was treated as the primary variable governing the volume fraction in the validation model. Accordingly, a lower volume fraction corresponds to a larger folding angle, whereas a higher volume fraction corresponds to a smaller folding angle.

**Figure 10:**
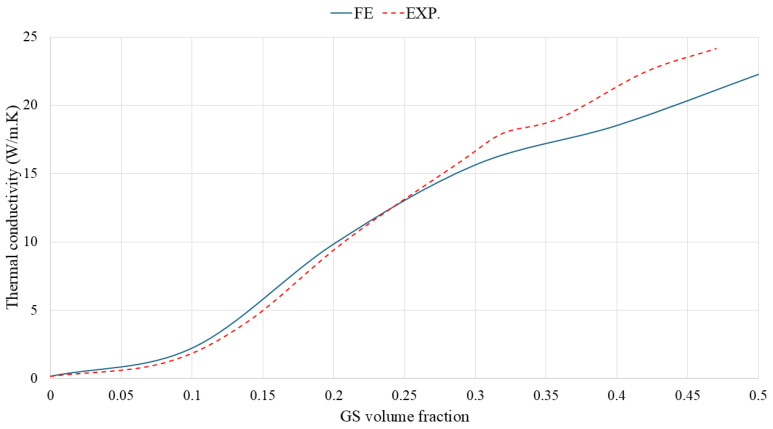
Experimental and FE-predicted values for the through-thickness thermal conductivity of the EP/GS composite at different graphite sheet volume fractions.

The FE model was therefore validated by comparing the predicted through-thickness thermal conductivity of the EP/GS composite with the experimental results reported by Li et al. [25]. As shown in Figure 10, both the FE and the experimental results exhibit the same overall trend, where the thermal conductivity increases continuously with increasing graphite sheet volume fraction. This agreement confirms the ability of the developed FE model to capture the heat transfer behavior of the folded composite structure.

At low graphite sheet volume fractions, the FE model slightly overestimated the experimental values, whereas at higher volume fractions, the FE predictions became lower than the experimental data. This crossover indicates that the discrepancy cannot be attributed to a single source, such as interfacial thermal resistance or voids alone. Rather, it suggests that the simplified periodic unit-cell model does not fully capture volume-fraction-dependent microstructural changes in the experimental composite. At higher graphite contents, additional graphite–graphite contacts, local stacking, compression-induced alignment, or nonuniform conductive pathways may contribute to the measured conductivity. Since these features were not explicitly characterized or included in the FE model, the validation should be interpreted as indicating agreement in the overall trend and order of magnitude, rather than a complete quantitative prediction across all volume fractions.

### 3.2. Effects of Graphite Sheet Volume Fraction and Folding Angle

**Figure 11:**
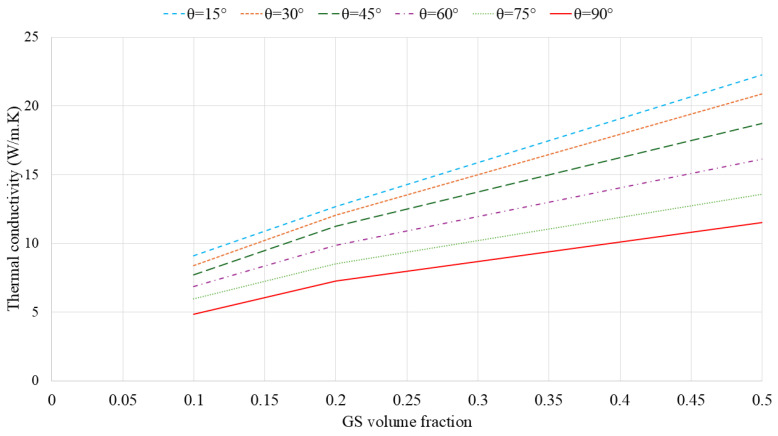
Composite through-thickness thermal conductivity as a function of the graphite sheet volume fraction at different folding angles.

Figure 11 shows the through-thickness thermal conductivity of the EP/GS composite as a function of graphite sheet volume fraction at different folding angles. For all folding angles, the thermal conductivity increased with increasing volume fraction. This behavior was expected because graphite sheets possess a significantly higher thermal conductivity than epoxy resin; therefore, increasing the sheet content enhances the formation of conductive heat transfer pathways through the composite thickness. The obtained results also demonstrated that the folding angle imparted a significant effect on the thermal conductivity. At the same graphite sheet volume fraction, lower folding angles resulted in a higher through-thickness thermal conductivity. For example, at a volume fraction of 0.5, the thermal conductivity decreased from 22.27 W m^−1^ K^−1^ at *θ* = 15° to 11.52 W m^−1^ K^−1^ at *θ* = 90°. This indicates that higher folding angles reduced the efficiency of heat conduction through the composite, and can be explained by considering the orientation of the graphite sheets relative to the heat flow direction. Specifically, at smaller folding angles, the graphite sheet structure is more favorably aligned in the through-thickness direction, allowing heat to be transferred via shorter, more continuous graphite pathways. In contrast, larger folding angles increase the tortuosity of the heat-transfer pathway and reduce the contribution of the high in-plane thermal conductivity of the graphite sheets. Consequently, the effective thermal conductivity decreased with increasing folding angle. Additionally, the influence of the folding angle was more pronounced at higher volume fractions, indicating that the presence of a greater amount of graphite further enhances the importance of conductive network orientation. At low graphite sheet volume fractions, the epoxy matrix dominated the overall thermal resistance, whereas at high volume fractions, the graphite network was the main pathway for heat conduction.

### 3.3. Effect of the Geometrical Ratio, E/2A

**Figure 12:**
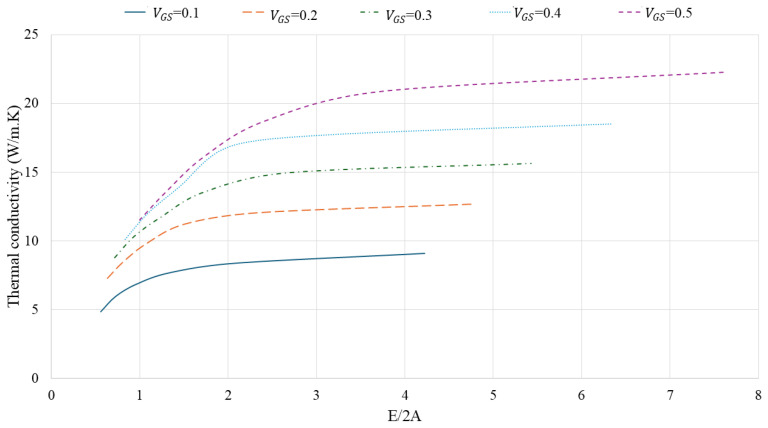
Composite through-thickness thermal conductivity as a function of *E/2A* at different graphite sheet volume fractions.

Figure 12 shows the variation in the through-thickness thermal conductivity as a function of the geometrical ratio (*E*/2*A*) at different graphite sheet volume fractions, revealing that thermal conductivity increases as *E*/2*A* increases for all volume fractions. This indicates that the geometry of the folded graphite sheets plays an important role in controlling the thermal performance of the composite. A higher *E*/2*A* ratio represents a taller and narrower folded structure, which improves the alignment of the graphite sheets in the through-thickness heat flow direction. Consequently, the lateral heat-flow deviation is reduced, and direct conduction is promoted through the graphite network. However, the increase in thermal conductivity became less steep at higher *E*/2*A* values, suggesting that the benefit of increasing the aspect ratio gradually approached a limiting value. This behavior indicates that beyond a certain geometry, further improvement in the graphite alignment produces only a modest increase in thermal conductivity. Notably, the effect of *E*/2*A* was stronger at higher graphite sheet fractions since larger quantities of graphite provide a more developed conductive network, rendering the composite more sensitive to changes in graphite orientation and geometry. Therefore, folded geometry optimization is particularly important when the composite possesses a high graphite sheet volume fraction.

### 3.4. Effect of Graphite Sheet Thickness

**Figure 13:**
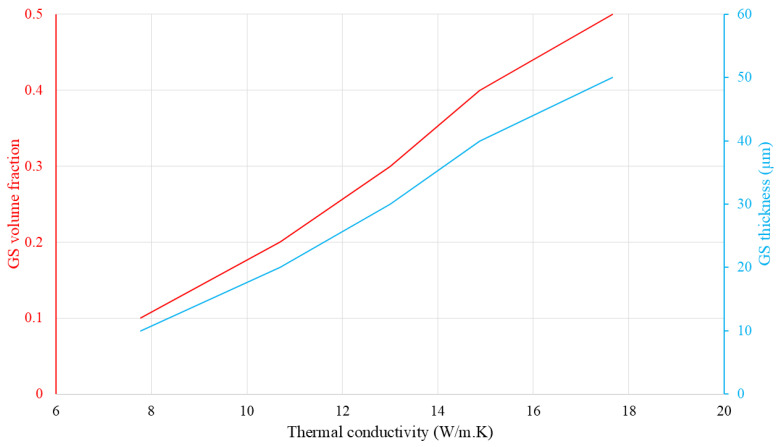
Composite through-thickness thermal conductivity as a function of graphite sheet volume fraction and thickness.

Figure 13 illustrates the relationship between the through-thickness thermal conductivity, graphite sheet volume fraction, and graphite sheet thickness. As shown, increasing the sheet thickness from 10 to 50 μm increased the thermal conductivity of the EP/GS composite from 7.78 to 17.66 W m^−1^ K^−1^. This improvement was primarily attributed to the larger conductive cross-sectional area provided by the thicker graphite sheets, which reduced the thermal resistance of the graphite network. Additionally, increasing the sheet thickness increased the volume fraction, further improving the thermal conductivity. Indeed, thicker graphite sheets provide more effective heat transfer channels and reduce the relative contribution of the low-conductivity epoxy phase. Nevertheless, this enhancement was less pronounced than that achieved by optimization of the folding angle, indicating that the orientation and arrangement of the graphite sheets have a greater impact on the through-thickness thermal performance than the thickness alone.

### 3.5. Comparison Between the Effects of the Folding Angle and Graphite Sheet Thickness

**Figure 14:**
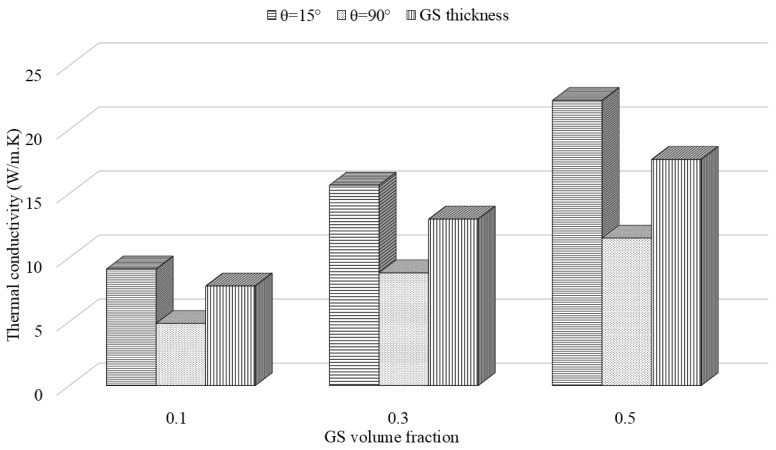
Comparison between the effects of the folding angle and the graphite sheet thickness at different volume fractions.

Figure 14 compares the effects of the folding angle and graphite sheet thickness at selected volume fractions. It can be seen that a smaller folding angle (e.g., 15°) produces a greater improvement in thermal conductivity than an increase in sheet thickness. However, the improvement in thermal conductivity with increasing thickness was greater than that at high folding angles (e.g., 90°). This confirms that the orientation of the graphite sheets plays a key role in controlling heat transfer through the composite thickness. At the same volume fraction, a smaller folding angle allows the high in-plane thermal conductivity of the graphite sheets to contribute more effectively to through-thickness conduction. In comparison, increasing the GS thickness improves the conductive area, but does not necessarily improve the alignment of the heat transfer pathway. Therefore, while both parameters enhance thermal conductivity, the folding angle imparts a more pronounced effect, as it directly controls the direction and continuity of the graphite conduction network.

Overall, the results demonstrated that the through-thickness thermal conductivity of the EP/GS composite was governed by a combination of the folding angle, geometrical ratio, graphite sheet volume fraction, and sheet thickness. Among these parameters, the volume fraction and folding angle exhibited the most significant effects. Specifically, increasing the GS volume fraction improved the thermal conductivity by increasing the amount of highly conductive filler within the epoxy matrix. However, the effectiveness of this increase strongly depended on the folding angle and the resulting orientation of the graphite sheets. The highest thermal conductivity was achieved when the composite possessed both a high volume fraction and a low folding angle, since this combination produced a more continuous and more uniformly aligned graphite network in the through-thickness direction. Conversely, larger folding angles reduced the thermal conductivity by increasing the tortuosity of the heat-transfer pathway and decreasing the contribution from the high in-plane conductivity of the graphite sheet. These findings highlight the importance of geometric design for optimizing the thermal performance of conductive polymers. Rather than relying solely on increasing graphite content or sheet thickness, the thermal performance can be significantly improved by controlling the folding angle and structural arrangement of the graphite sheets. Optimizing the architecture of the graphite sheet network is therefore essential in the development of high-performance EP/GS composites for thermal-interface material applications.

### 3.6. Semi-Empirical Thermal Conductivity Model

Based on the observed relationship among the graphite sheet volume fraction, folding angle, and through-thickness thermal conductivity, a semi-empirical model was developed to describe the effective thermal conductivity of the EP/GS composite. Specifically, this model was developed based on the FE simulation results and was fitted via regression analysis. Artificial intelligence was employed to clarify the wording and organization of the model description; all equations, fitting parameters, and interpretations were independently verified by the authors. This model combined the contribution of the epoxy matrix with the orientation-dependent contribution of the anisotropic graphite sheets. Since the graphite sheets exhibited significantly different in-plane and through-thickness thermal conductivities, their effective contribution in the through-thickness direction was expressed as a function of the folding angle, as follows:

(9) $$k_{GS,\theta }=K_{GS∥}{cos}^{2}\left(\frac{\theta }{2}\right)+K_{GS⊥}{sin}^{2}\left(\frac{\theta }{2}\right)$$

where $K_{GS∥}=\;$130.35 W m^−1^ K^−1^ is the in-plane thermal conductivity of the graphite sheet, $K_{GS⊥}=$ 10 W m^−1^ K^−1^ is its through-thickness thermal conductivity, and *θ* is the folding angle. This expression accounts for the varied contributions of the highly conductive in-plane direction of the graphite sheet as its orientation changes relative to the heat flow direction.

The effective through-thickness thermal conductivity of the composite was described using a modified rule of mixtures approach:

(10) $$K_{CompT}=K_{EP}V_{EP}+\eta V_{GS}^{n}K_{GS,\theta }$$

where $K_{CompT}$ is the through-thickness thermal conductivity of the composite and $K_{EP}=$ 0.18 W m^−1^ K^−1^ is the thermal conductivity of the epoxy matrix. Additionally, $V_{EP}$ is the epoxy resin volume fraction, $V_{GS}$ is the graphite sheet volume fraction, η is an efficiency coefficient, and n is a volume-fraction exponent. By fitting the model to the FE results, the values of η and n were determined to be 0.239 and 0.560, respectively. Therefore, the final semi-empirical model can be written as:

(11) $$K_{CompT}=K_{EP}V_{EP}+0.239V_{GS}^{0.560}\left[K_{GS∥}{cos}^{2}\left(\frac{\theta }{2}\right)+K_{GS⊥}{sin}^{2}\left(\frac{\theta }{2}\right)\right]$$

In this expression, the coefficient η = 0.239 represents the effective utilization of the intrinsic graphite sheet conductivity within the folded composite structure. Although graphite sheets possess high in-plane thermal conductivity, their full conductivity is not completely transferred to the composite because of the geometric tortuosity, discontinuity of the heat-transfer pathway, and thermal resistance at the epoxy/graphite interface. This coefficient therefore acts as an efficiency factor that scales the contribution of ideal graphite to its effective contribution to the composite properties. Additionally, the exponent n = 0.560 describes the nonlinear dependence of the thermal conductivity on the graphite sheet volume fraction. Since the value of n is lower than unity, the relationship between graphite content and thermal conductivity is sublinear. This indicates that although increasing the graphite volume fraction promotes heat transfer, the resulting improvement is not directly proportional to the added graphite content. This behavior may be attributed to the increasing complexity of the folded graphite network, changes in sheet orientation, and interfacial- and matrix-related thermal resistances.

**Figure 15:**
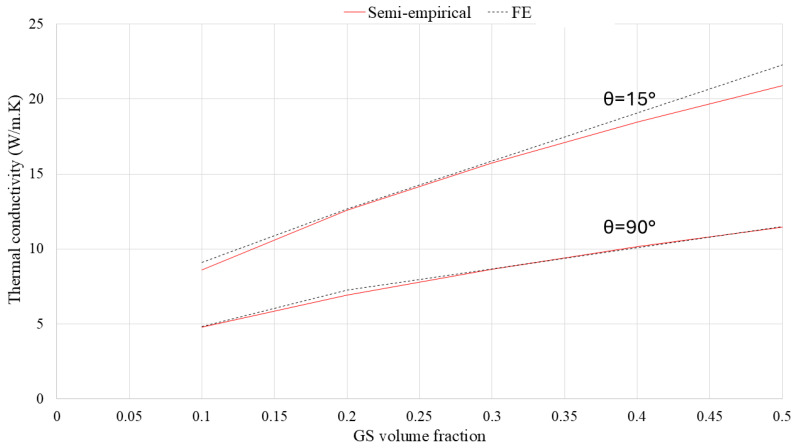
Comparison between the FE and semi-empirical results at different folding angles and graphite sheet volume fractions.

Importantly, the developed model corresponded well with the FE simulations, yielding a coefficient of determination of R^2^ ≈ 0.989. Figure 15 shows a comparison of the FE and semi-empirical results for folding angles of 15° and 90°. This indicates that the proposed equation successfully captures the combined effects of the graphite sheet volume fraction and the folding angle on the through-thickness thermal conductivity of the EP/GS composite. The model also supports the trends observed in the parametric study: increasing $V_{GS}$ enhances thermal conductivity, whereas increasing the folding angle reduces the effective contribution of the high in-plane conductivity originating from the graphite sheets. Thus, the current semiempirical model provides a compact FE-calibrated correlation for representing the numerical trends in the thermal performance of folded graphite sheets/polymers observed in this study.

## 4. Conclusions

This study investigated the through-thickness thermal conductivity of epoxy resin/graphite sheet (EP/GS) composites using finite element (FE) modeling. A representative unit cell was developed to describe the periodically folded zigzag graphite sheet structure, and steady-state thermal analysis was used to predict the effective thermal conductivity. The FE model was validated using experimental data from the literature, and good agreement was obtained, confirming the reliability of the numerical approach. Increasing the volume fraction of the graphite sheets enhanced the through-thickness thermal conductivity by forming more effective conductive pathways in the epoxy matrix. However, this improvement was strongly dependent on the folding angle of the graphite sheets. Lower folding angles produced higher thermal conductivities, as the graphite sheets were more effectively aligned in the through-thickness heat-flow direction. At a graphite sheet volume fraction of 0.5, the thermal conductivity decreased from 22.27 W m^−1^ K^−1^ at a folding angle of 15° to 11.52 W m^−1^ K^−1^ at 90°. Moreover, the geometrical ratio (*E*/2*A*) and graphite sheet thickness also influenced thermal performance. Specifically, increasing *E*/2*A* improved sheet alignment, while increasing the graphite sheet thickness from 10 to 50 µm enhanced thermal conductivity from 7.78 to 17.66 W m^−1^ K^−1^. Nevertheless, reducing the folding angle had a more pronounced effect than increasing the thickness, indicating that graphite sheet orientation is the dominant factor governing heat transfer. Finally, a semi-empirical model was developed, exhibiting good agreement with the FE results. Overall, these findings demonstrate that optimization of the folded-graphite-sheet architecture is essential for the development of high-performance polymer-based thermal interface materials.

Despite these findings, the FE model was based on an idealized, periodically repeated unit-cell geometry, in which the folded graphite sheets were represented by simplified straight-line features. In practice, however, folded graphite sheets may exhibit local irregularities, waviness, misalignment, defects, and non-uniform spacing, all of which can influence the formation of conductive pathways and the resulting thermal conductivity. Therefore, future work should consider more realistic graphite sheet geometries and manufacturing-induced imperfections to further improve the accuracy of the numerical predictions.

## Figures and Tables

**Table 1:** Material properties of the constituent materials of the EP/GS composite.

Material	Density	Thermal Conductivity	Source
	(kg m^−3^)	(W m^−1^ K^−1^)	
Epoxy resin	1110	0.18	[29]
Graphite sheet	600	130.35 (in-plane) 10 (through-thickness)	[30]

## Data Availability

The data presented in this study are available on request from the corresponding author. The data are not publicly available due to the ongoing nature of the research.

## Abbreviations

The following abbreviations are used in this manuscript:

TIM	Thermal interface material
EP/GS	Epoxy resin/graphite sheet
PMC	Polymer matrix composite
FE	Finite element
RVE	Representative volume element
